# Bioactive Compounds Produced by Endophytic Bacteria and Their Plant Hosts—An Insight into the World of Chosen Herbaceous Ruderal Plants in Central Europe

**DOI:** 10.3390/molecules29184456

**Published:** 2024-09-19

**Authors:** Piotr Drożdżyński, Natalia Rutkowska, Magdalena Rodziewicz, Olga Marchut-Mikołajczyk

**Affiliations:** Institute of Molecular and Industrial Biotechnology, Faculty of Biotechnology and Food Sciences, Lodz University of Technology, Stefanowskiego 2/22, 90-537 Lodz, Poland; natalia.rutkowska@dokt.p.lodz.pl (N.R.); rodziewicz.mag@gmail.com (M.R.); olga.marchut-mikolajczyk@p.lodz.pl (O.M.-M.)

**Keywords:** synanthropic herbaceous plants, endophytic bacteria, biologically active compounds, biosynthetic gene clusters

## Abstract

The natural environment has been significantly impacted by human activity, urbanization, and industrialization, leading to changes in living organisms and their adaptation to harsh conditions. Species, including plants, adapt to these changes by creating mechanisms and modifications that allow them to survive in harsh environments. Also, endophytes, microorganisms that live inside plants, can support plant growth and defense mechanisms in these conditions by synthesizing antimicrobial secondary metabolites. What is more, endophytes produce bioactive metabolites, including alkaloids, amines, and peptides, which play a crucial role in the relationship between endophytes and their host organisms. Endophytes themselves benefit from this by creating a stable environment for their survival and development. The aim of this review is to gain insight into endophytic bioactive metabolites from chosen synanthropic ruderal plants. Industrial activities release pollutants like heavy metals, by-products, and waste, which challenge living organisms and require adaptation. Synanthropic plants, where endophytes are abundant, are particularly valuable for their bioactive compounds, which are used in agriculture and medicine. This review presents, among others, endophytes of herbaceous ruderal plants from central Europe—*Chelidonium majus* L., *Urtica dioica* L., *Plantago lanceolata* L., *Matricaria chamomilla* L., *Equisetum arvense* L., *Oenothera biennis* L., *Silybum marianum* L., and *Mentha piperita* L.

## 1. Introduction

Over the years, the natural environment has undergone many changes, often as the result of human activity, its broadly understood development, and progressive urbanization. Because of the shrinkage of natural water bodies, wetlands, and the gradual drying out of soils near cities, the transformation of the economy and thus industrialization has not only caused a dramatic change in the conditions prevailing there but has also made it impossible for some maladjusted species of living organisms to continue to exist. Synanthrophization refers to the whole of human impact, including direct and indirect acts that result in a specific modification to the environment and a need for adaptation by the organisms that inhabit it. Species, including plants, with different degrees of effectiveness respond to these needs by creating various types of mechanisms and modifications that allow them to develop in ruderal areas. Metabolism changes caused by changing environmental conditions are often associated with the production of biologically active compounds. By adapting to new living conditions, such plants can displace other species, which is why they are often called weeds, but some of them turn out to have healing potential. The functioning of plants in difficult conditions can also be facilitated by microorganisms inhabiting the inter-tissue spaces of plants, which not only do not harm the host but often produce many substances that allow plants to survive in unfavorable environmental conditions.

## 2. Endophytes and Their Sources

According to Wilson’s 1995 definition [1], endophytes are fungi or bacteria which, for all or part of their life cycle, invade the tissues of living plants and cause unapparent and asymptomatic infections entirely within plant tissues but cause no symptoms of disease. These microbes are often found in intracellular and intercellular areas and vascular tissue and can colonize aerial parts or roots [2]. However, nowadays, it is clear that endophytic bacteria mostly derive from the rhizosphere environment [2]. The term “endophyte” has become a synonym for a “mutualist” or “commensalist” symbiont [2,3], and evolutionary endophytic bacteria are considered an intermediate group between saprophytic and pathogenic bacteria. Nevertheless, not all colonizers are harmless; some plant pests are often bacterial or fungal [4,5,6].

**Figure 1:**
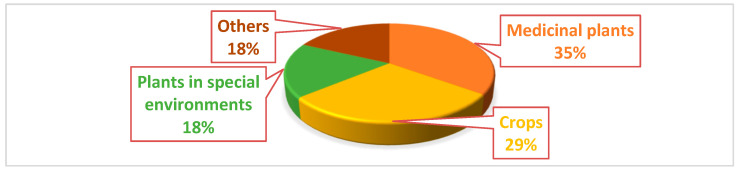
Examination of the ratio of biologically active strains from various sources that were evaluated for antibacterial activity based on research studies over the last decade [7,8].

Based on current studies, medicinal and agricultural plants are commonly prioritized for the isolation of endophytes. Furthermore, fungal endophytes garner greater scrutiny compared to bacterial endophytes. Figure 1 illustrates a comparison of the endophytic hosts, revealing that medicinal plants, crops, and plants in specialized environments have been extensively investigated for the purpose of identifying antimicrobial endophytes [7].

**Figure 2:**
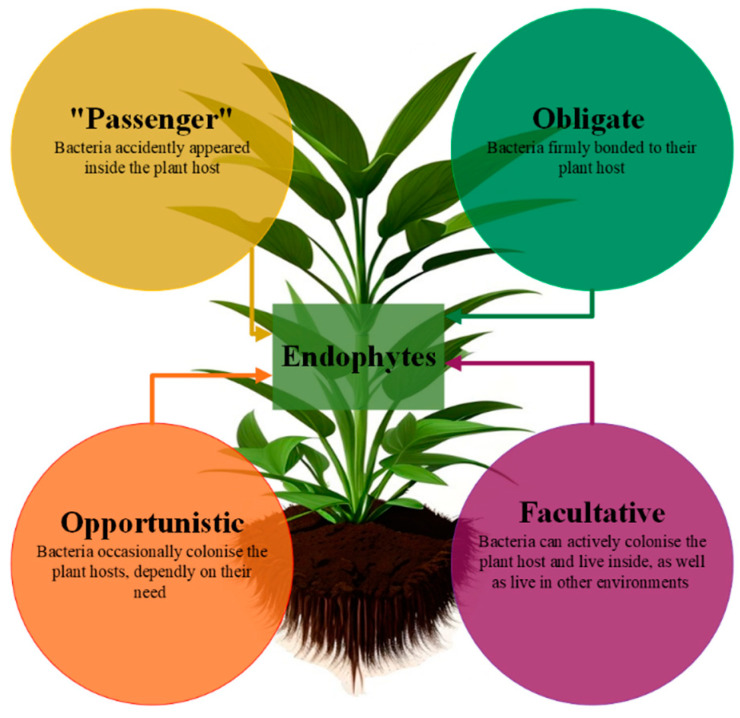
Lifestyle-based categorization of endophytic bacterial species [12,13].

Among the several division classifications suggested by Hardoim [9], endophytic microorganisms are classified as obligate, facultative, opportunistic, or “passenger”, based on the distinct internal mechanisms used to colonize a plant, as depicted in Figure 2 [9,10,11]. 

Opportunistic endophytes are microorganisms that invade plants to help them survive and thrive in harsh environments. The term “passenger endophyte” refers to microorganisms that inadvertently colonize a plant. Since obligatory endophytes are likely passed on through seeds instead of the rhizosphere, they cannot spread outside of plants. One the one hand, they depend on the plant’s metabolic activity for their survival and spread inside the plant by vertical colonization or via the action of a vector; on the other hand, they are vital to the plant’s ability to maintain its internal environment [4,12,13,14]. Facultative endophytes can live both within plants and outside of them; they are found living unrestrictedly in the soil, and when the chance presents itself, they will colonize inside plants through coordinated infection. The rhizosphere is the location where this process takes place.

A multitude of studies have conclusively shown that endophytic communities exhibit high levels of diversity, with nearly all of them being facultative. A number of researchers, such as Hallmann et al. (1997), Afzal et al. (2019), Santoyo et al. (2016), Surjit et al. (2014), and Rosenblueth and Martinez-Romero (2006), have presented data about the diverse range of endophytic communities [4,14,15,16,17]. Stochastic occurrences, such as open wounds along the root hairs, are what allow passive endophytes to colonize a plant. Passive endophytes do not actively strive to colonize the plant. They are referred to as associated bacteria since they are attached to the surface of the roots. In addition, bacteria that live in the rhizosphere (rhizobacteria) that are closely linked to the plant roots also occur. Endophytes that have a passive life strategy are less competitive than other endophytes because the cellular machinery that is necessary for plant colonization is absent from their cells [2,16,18]. Passive penetration may occur at the root tip, root hairs, or elongation zone level and at cracks such as those occurring at root emergence sites, but in general, many endophytes are equipped with cell-wall-degrading enzymes such as cellulases, which are probably needed for efficient plant colonization and spreading within plant tissues [19,20]. Structures such as stomata, hydathodes, lenticels, nectarines, and nectar glands, as well as tissue damage that is caused by the attack of insects or broken trichomes, are also entry routes for soil microbiota [21]. These colonizers are capable of establishing populations both intercellularly and intracellularly [22,23,24].

## 3. Endophytic Plant Colonization Mechanisms 

The process of root colonization can be categorized into two primary phases. During the early phase, bacteria attach to the root cells and then reproduce, resulting in their widespread distribution throughout the whole surface. In this phase, there is a rapid increase in the number of microorganisms in habitats that have limited natural boundaries.

Within the rhizosphere, a specific cluster of microbes demonstrates a greater capacity to use root secretions as a carbon and energy substrate. This grants them a greater capacity to rival other bacterial species with enhanced effectiveness [24]. The colonization process begins with the identification of particular plant root secretions by bacteria. Certain bacteria demonstrate chemotaxis, a phenomenon in which they are drawn towards the origin of secretions due to their recognition of certain chemical compounds, such as phenolic acids or amino acids. The chemotactic response to certain components in root secretions is crucial for bacteria to establish colonization in plants [17]. Moreover, research has revealed that various plant tissues can harbor distinct endophyte communities with varying compositions [25].

The *Pseudomonas stutzeri* strains PPS96, PSR2, and PSR21, which showed the greatest ability to colonize wheat roots, were found to utilize p-hydroxyphenylacetic acid, bromosuccinic acid, benzoic acid, methyl pyruvate, N-acetyl-D-glucosamine, and D-trehalose as their sole carbon and energy sources. In contrast, *Pseudomonas stutzeri* P221, PPS16, PSR25, PSR67, PSR79, PSR81, PSR82, and PSR120 strains that had a restricted ability to colonize roots did not show this capability [26].

Some parts of plants on land can be colonized by different microbes. In addition, the surfaces of stems and leaves can potentially release exudates that attract bacteria.

During the process of phyllosphere colonization, bacterial strains first attach to the surface of the leaf and then spread out in a random fashion. Specific bacteria have the capacity to penetrate leaf tissue via natural openings, such as stomata and hydathodes, therefore influencing their local environment. Yaron and Römling (2014) found that bacterial strains in this specific region reproduce and create a fragile biofilm. However, a portion of these strains have the capability to infiltrate the leaf tissue and establish themselves as endophytes [27]. James et al. (2001) identified the stomata as the entry route for the colonization of the *Gluconobacter diazotrophicus* strain in sugarcane [28]. Regrettably, the presence of UV radiation, nutritional deficiency, and desiccation often hinders the colonization of leaf surfaces [29].

Passive mechanisms can also aid in the process of host invasion [9,30]. Soil-dwelling bacteria can unintentionally become endophytic by establishing themselves in natural wounds or when their roots are invaded by a parasite, among other ways. These bacteria are categorized as guest endophytes and commonly exist only in the root cortical tissue. Like passenger endophytes, opportunistic endophytes are also restricted to particular plant tissues, such as the root cortex. It is proposed that competent endophytes possess all the traits of opportunistic endophytes and demonstrate a significant level of adaptability to the plant environment. These organisms possess the capability to penetrate specific plant tissue, such as vascular tissue, and spread across the entire plant. They accomplish this by regulating plant metabolism, enabling them to coexist with the plant host even in significant quantities [30]. 

Horizontal transmission appears to be the main method of dispersion among endophytic species. Endophytes propagate through the production of spores, which are then dispersed by insects and wind. Endophytes can be horizontally transmitted, meaning they can spread from one infected plant to uncolonized ones. Endophytes that are transmitted by seeds and vertically inherited are commonly found as epiphytes, suggesting that endophytes may also establish colonies in the environments around host plants [31].

Endophytic bacteria can infect plants by vertical transmission, which refers to the transfer from the parent to successive generations. Specific endophytes that inhabit the seeds are crucial for guaranteeing their transfer to the progeny. This is also applicable to endophytes that travel together with seedlings of fruit trees, tubers, and rootstocks. The bacteria in question occupy a favorable habitat, and it is likely that other microorganisms in the rhizosphere may undergo slight changes in response to their presence [32].

When examining endophytes that come from the rhizosphere, it is crucial for them to successfully establish colonization in a new host by entering through the root or another portion of the plant.

### 3.1. Physiological Mechanisms

The primary mechanisms by which plants undergo bacterial colonization include the modulation of the root exudate composition and the activation of an innate immune response. The colonization of the plant is influenced by both random events and predictable bacterial factors. The process of colonizing plant roots entails the formation of microcolonies on the root surface. Opportunistic endophytes possess distinct characteristics that enable them to colonize roots, such as a chemotactic response; multiple studies [17,33] have demonstrated that endophytes exhibit a chemotactic response when exposed to root exudates from their host plants. Endophytes and other beneficial microorganisms actively acquire or distinguish the biomolecules that are abundant in root exudates. Exudates possess a prodigious supply of water and nutrients, which magnetize microorganisms chemotactically. A wide variety of plants facilitate endophytic contact with potential plant hosts via their flavonoid pigments, which function as chemoattractants [26,34]. The study conducted by Bacilio-Jimenez et al. (2003) revealed the presence of chemotactic mechanisms. The researchers observed that the rice root exudates were predominantly composed of glucose, arabinose, mannose, and galactose, as well as amino acids like histidine, proline, alanine, and glycine. Within the initial two weeks after the plants were planted, there was a significant rise in and variety of carbohydrates and amino acids [35].

The chemotactic response of endophytic isolates of *Corynebacterium flavescens* and *Bacillus pumilus* towards the components of rice root exudates exhibited a significantly higher magnitude, ranging from 3.9 to 5.1 times, compared to the tested isolate *Azospirillum brasiliense* [35]. The chemotaxis of the endophytic isolates mentioned was 2.2 to 2.8 times higher compared to the second strain, *Bacillus* sp. Potential endophytes display a major advantage by being able to use various parts of root secretions throughout the process of root colonization [26].

To preferentially attract the mutualistic microorganisms, especially the potential endophytes, plant roots release a variety of secondary metabolites, including sugars, amino acids, organic acids, flavonoids, phenolic compounds, and others [36]. The success of root colonization in rice and wheat by *Azorhizobium caulinodans* ORS571 and the endophytic *Serratia* sp. EDA2 was significantly enhanced in the presence of flavonoids, a category of metabolites that have also been implicated in the case of non-rhizobial endophytes [37,38].

What is more, the colonization of plant hosts depends on many factors and is affected by genotype, growth phase, physiological status, plant tissue type, taxon and strain type, agricultural practices, and environmental variables (temperature, water supply, and nutrients) [29]. 

### 3.2. Molecular Mechanisms

Molecular patterns also drive all stages of colonization. Several important genes found in bacterial genomes play a critical role in their ability to chemotax and migrate as well as adhere to the surface of roots and navigate throughout plants. The aforementioned discoveries were facilitated by the use of comparative genomics investigations and the examination of bacterial mutants, as extensively detailed by Pinski and coworkers [39]. In their 2002 work, de Weert and colleagues investigated the reaction of bacterial strains belonging to the species *Pseudomonas fluorescens* to chemotaxis while colonizing tomato roots. Wild strains and mutant strains were utilized, wherein mutations were introduced in the *cheA* gene, which is responsible for the flagella-mediated movement towards chemical attractants in tomatoes. The results of their experiments validated that the faulty *cheA* gene significantly reduced the competitive colonization of tomato root tips. All *cheA* mutants demonstrated a 10- to 1000-fold reduction in their ability to colonize the root tip of the vegetable compared to their wild counterpart after the seedlings received an inoculation with a 1:1 mixture [40]. In 2016, Rossi and colleagues investigated the *Azospirillum brasilense* strain and its capacity to adhere to maize roots. Cells of *A. brasilense* were rendered immotile as a consequence of alterations in the polar flagellum assembly caused by mutations in the *flmA* or *flmB* genes. Moreover, these alterations also impacted the competitive capacity of A. brasilense cells to attach to maize roots and generate lipopolysaccharides and exopolysaccharides [41]. Attachment and biofilm formation of new colonizers is the next step during colonization. Duque et al. employed a hybrid methodology using in silico algorithms and mutagenesis techniques to examine the genetic factors that influence the surface attachment of *Pseudomonas putida* strain KT2440. An analysis revealed that the genes encoding the synthesis of the major adhesion proteins LapA and LapF and flagellar proteins were highly conserved in all the sequenced strains of *P. putida*. Disabling these genes resulted in a reduced capacity to compete in colonizing maize roots [42].

In brief, the colonization of plant roots by bacteria is a progressive and multifaceted process that initiates in the rhizosphere with the migration of bacteria towards the root, followed by their adherence and subsequent development of a biofilm. Thus far, the majority of the genetic factors influencing bacterial colonization have been evaluated in experimental investigations involving a single strain, conducted in sterile environments. However, bacterial colonization can be influenced by both plant–bacteria and bacteria–bacteria interactions. Unfortunately, the tremendous variety and complexity of the root-associated microbiota hinder the characterization of the molecular pathways [43].

This reaction allows them to first colonize the rhizoplane and then infiltrate the interior tissues of the plant through fissures that occur at the points where lateral roots form and at the tips of the roots. The main cause of this phenomenon is the presence of methyl-accepting chemotaxis proteins (MCPs), which are sensors located in the bacterial membrane. These sensors detect chemicals in the surrounding environment and steer the bacteria towards attractive substances or away from repellents [35]. One study found that disabling the Hsero_3720 gene, which encodes MCPs, in the mutant strain *Herbaspirillum seropedicae* SmR1 resulted in a 50% decrease in its capacity to attach to maize roots compared to a non-mutated strain [44].

## 4. Interaction between Plants and Endophytic Microorganisms

The relationship between an endophyte and a plant is considered symbiotic since both organisms benefit from this partnership. The co-evolutionary process results in the existence of an intimate relationship between the endophyte and its host, and this interaction is caused by changes at the cellular and molecular levels that affect the development of the plant, namely, improvements in plant growth and health through a variety of processes, including nitrogen fixation, the synthesis of plant hormones (phytohormones) and vitamins, and the enhancement of nutrient, mineral, and water uptake, as well as the induction of stress resistance [45]. Stress can be caused by abiotic factors such as osmotic stress as well as exposure to heavy metals and xenobiotic compounds. Endophytes can also accelerate digestion in plants and phosphorus solubilization, which provide resistance to biotic factors. Microbial interactions, which are advantageous to the host plant, are necessary for the development of potential endophyte colonies on the surface of the root. Through a mechanism referred to as cross-talk of signal molecules, an endophyte colonizes a host plant, which subsequently initiates communication with the invading endophyte. Following the initial colonization, certain endophytes have the ability to distribute themselves across various regions of the plant by utilizing the vascular tissues, a phenomenon referred to as systemic dissemination. Johnston-Monje and Raizada (2011) used endophytes that were labeled with green fluorescent protein (GFP) to demonstrate how these microorganisms move from seeds to the plant’s roots and tissues. Additionally, they injected endophytes into stems and observed how they moved into the roots and rhizosphere. The results of this study imply that there may be a continuous flow of organisms throughout the root microbiome [46,47]. The relationship between plants and endophytic bacteria has a substantial impact on plant growth, health, development, yield, and soil quality. These interactions also give many benefits to the species involved and have numerous positive effects on the integrity and sustainability of agroecosystems. Then, they emphasize the relevance of the increase in growth, plant biomass, dry matter yield, or grain yield, thus contributing to a rise in agricultural income.

Endophytic microorganisms may outcompete invading pathogens through a variety of processes, including niche occupation through competition for space, resources, and physical niches of the rhizosphere or rhizoplane and endophytic tissues. Some beneficial endophytic bacteria can secrete antibiotics and lytic enzymes that enable the inhibition of various pathogens. Moreover, the artificial inoculation of endophytic microbes into plants can considerably minimize the attack of disease-causing factors such as fungi, bacteria, viruses, insects, and nematodes, thereby biologically controlling these pathogens [14,48].

These microorganisms may outcompete invading pathogens through a variety of processes, including niche occupation through competition for space, resources, and physical niches of the rhizosphere or rhizoplane and endophytic tissues. Some beneficial endophytic bacteria can secrete antibiotics and lytic enzymes that enable the inhibition of various pathogens [49,50].

## 5. Surface Sterilization of Plant Material

Endophyte isolation is a crucial step in the study of endophytic bacteria and fungi. Endophytic microorganisms, such as bacteria and fungi, are isolated in order to describe and explore their population dynamics and diversity. In addition, they are isolated in order to utilize their microbial inoculants to promote plant development and health and to obtain novel physiologically active secondary metabolites [51]. The crucial part of the isolation of endophytic microorganisms is the surface sterilization of the plant material. Table 1 lists the most frequent and widely recognized sterilizing methods. The success of surface sterilization is dependent on a variety of characteristics, including the sterilizing agent employed, the resistance of the plant tissue samples to the sterilizing agents, and the time and concentration of the sterilizing agent [52].

In terms of isolating endophytic bacteria, the sterilizing agent is one of the most influential elements. One of the most prevalent sterilizing agents is ethyl alcohol (EtOH), an amphiphilic chemical that damages cell membranes, denatures proteins, and dissolves lipids inside the cell sap. As a result of ethanol application, the cell walls leak, and it dissolves important cell organelles, which eventually leads to the death of cells. The commonly used concentration oscillates between 60 and 90%. Higher concentrations are also used and are considered efficient against spores. Equally common to ethanol as a sterilizing agent is sodium hypochlorite, which has powerful oxidizing characteristics and a wide antibacterial spectrum [53,56,57,60,82]. Sodium hypochlorite in a solution forms hypochlorous acid, which can permeate microbial cell walls and membranes, causing protein denaturation and lipid breakdown inside the cell, leading to cell death. The concentrations used for surface sterilization typically range from 0.5% to 5% [83,84].

Occasionally, mercuric chloride is used for sterilization because it is efficient even at low concentrations. Mercury chloride, however, poses health risks to humans and results in the creation of toxic waste. As a consequence, there is a high potential for negative effects on the environment and human health when it is used [85]. It is unusual for a single sterilizing chemical to be sufficient for the surface sterilization of plant samples. In order to achieve successful sterilization results, it is best to use a combination of two or more chemicals. Common formulations contain 75% ethanol and 2% sodium hypochlorite [57,60,82]. This combination is useful in most types of isolation techniques. Some cases call for the use of surface-active agents to help with the process of sterilizing surfaces. Gupta et al. (2015) isolated endophytic bacteria using surface sterilization. Surface sterilization was carried out first by washing in 1% Savlon for 5 min. Then, the leaves and roots were treated with 0.1% mercuric chloride for surface sterilization [86]. Gohain et al. (2015) conducted a surface sterilization of plant samples (leaves, stems, and roots) from healthy plants in Gibbon. The samples were rinsed in 0.1% Tween 20 for 30 s, then surface-sterilized by immersing them sequentially in 70% ethanol for 2 min followed by 1% sodium hypochlorite for 3 min, 70% ethanol for 2 min, and then three washes with sterile distilled water for 5 min [87]. Qin et al. (2009) carried out a ‘five-step’ surface sterilization procedure consisting of a 3 min wash in sodium chlorate (5% NaClO_3_), followed by a 10 min wash in sodium thiosulfate (2.5% Na_2_S_2_O_3_), followed by a 3 min wash in 75% ethanol, followed by a 10 min wash in sodium bicarbonate (10% NaHCO_3_), and a final wash in sterile water [80]. Coombs and Franco (2003) conducted a three-step surface sterilization procedure of plant roots as follows: a 60 s wash in 99% ethanol, followed by a 6 min wash in 3.125% NaOCl, another 30 s wash in 99% ethanol, and a final rinse in sterile reverse-osmosis-treated (RO) water [88].

There are also methods that avoid surface sterilization, such as vacuum or pressure extraction [14]. However, these techniques are not recommended for delicate, non-woody plant tissues, as they will collapse under vacuum. Gardner et al. (1982) and Bell et al. (1995) effectively isolated endophytic xylem-residing bacteria from the roots of a citrus tree in Florida and a grapevine tree in Nova Scotia [89,90]. Scholander et al. (1965) introduced a second method for extracting plant sap using a Scholander pressure bomb. This technique was utilized to successfully isolate endophytic bacteria from roots. The Scholander pressure bomb is less labor-intensive than trituration, which is an advantage [91]. However, limitations of this technique were encountered when working with immature, fleshy cucumber tissues [92].

## 6. Bioactive Metabolites from Endophytic Bacteria

**Figure 3:**
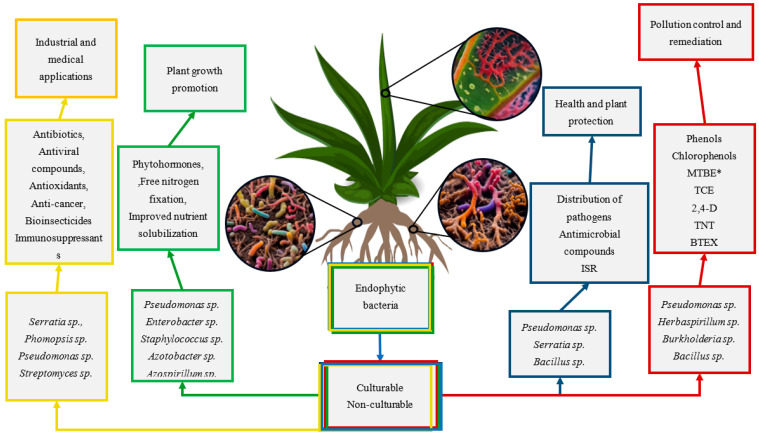
Compounds with biological activity produced by endophytic bacteria and their potential use in biotechnology [45,95,96,97]. * MTBE—tert-butyl methyl ether; TCE—1,1,1-trichloroethylene; 2,4-D—2,4-dichlorophenoxyacetic acid; TNT—2,4,6-trinitrotoluene; BTEX—benzene, toluene, ethylbenzene, and xylene isomers.

Endophytes, as microorganisms inhabiting the interior of plant tissues, must adapt not only to the environmental conditions there but also to a variety of plant defense mechanisms. Their strategy has its foundation in the production of a wide range of bioactive compounds that support the growth of the host organism or defend it from adverse factors. The biochemistry of plants and microbes relies on many pathways encompassing numerous intermediate metabolites, all of which hold significant importance. Frequently, individual bioactive compounds exhibit many functions, resulting in a diverse range of relevance. These compounds engage in interactions that give rise to complex, multi-level connections, hence precluding the occurrence of singular outcomes for a given bioactive chemical [93,94]. The production of metabolites by endophytes serves several crucial functions, including supporting development in host tissues, supplying essential chemicals for survival, and promoting successful plant colonization, as shown in Figure 3 [45]. Endophytic bacteria and actinomycetes have a crucial function in the synthesis of bioactive chemicals (alkaloids, steroids, terpenoids, peptides, polyketones, flavonoids, quinols, phenols, and the natural insecticide azadirachtin). 

Table 2 gives a compilation of the bioactive chemicals synthesized by endophytic bacteria obtained from synanthropic herbaceous plants found throughout Europe.

The beneficial impact exerted by endophytes on the plant host results in plants refraining from actively eliminating them. Endophytic metabolites that have nitrogen in their chemical structures, such as alkaloids, amines, non-protein amino acids, peptides, glucosinolates, alkamides, and cyanogenic glycosides, play a significant role in the natural nitrogen cycle process and the mutual relationship between endophytes and their host organisms. Several of these factors are crucial for the survival of plants, such as amino acids, which are the fundamental components of plant proteins and enzymes, and tetrapyrrole structures, which are crucial components of chlorophyll molecules [34,129,130,131]. Moreover, a significant proportion of these molecules have medicinal properties and have been effectively employed in the synthesis of pharmaceuticals, including treatments for presently incurable conditions such as cancer [132]. However, the functions of metabolites produced by endophytic microorganisms vary and depend on the structure of a given compound. Fungal endophytes are a prominent category of endophytic microorganisms that have garnered significant attention due to their production of bioactive chemicals. This recognition stems from their frequent isolation from plant tissues that possess therapeutic properties. However, despite a limited understanding of this topic, an increasing body of scientific research suggests that more classes of endophytic microbes possess the ability to generate useful metabolites [133].

Synanthropic plants are a particularly intriguing source of endophytes that produce bioactive metabolites. These types of plants inhabit environments that are subject to intense anthropological pressure; as a result, they are exposed to numerous negative factors associated with the growth of civilization. The presence of endophytes appears to be invaluable for these plants. The bioactive compounds that have been extracted from synanthropic herbaceous plants encompass several groups, including phenols, alkaloids, benzopyrenes, glycosides, flavonoids, terpenoids, non-protein amino acids, cyanogenic glycosides, and numerous others [32,134,135,136,137,138]. Certain chemicals with distinct characteristics and the potential for microbial synthesis are isolated and employed in commercial applications, such as agriculture, for their natural pesticide properties as well as in medicinal contexts for the treatment of diverse human ailments [137].

Industrial activities are responsible for the production of a wide range of pollutants, including heavy metals, by-products, and waste. These pollutants impose significant challenges on living organisms, compelling them to adapt to adverse conditions. The growth and development of plants in challenging conditions primarily rely on the synthesis of compounds that serve to inhibit the entry of detrimental substances into cells or enhance the tolerance range of individuals, thereby ensuring that their presence does not hinder plant development. Endophytes, being microorganisms with the ability to synthesize diverse secondary metabolites, significantly enhance and occasionally even permit adaptation and persistence in challenging environments resulting from anthropogenic activities. Minimizing the detrimental impacts of non-biodegradable heavy metals is of paramount importance. Endophytic microorganisms have demonstrated their use and substantial contribution in enhancing the efficacy of the phytoremediation process [95,139,140]. The occurrence of stress factors has a direct effect on the endophytic microorganism population in plant tissues. In the case of synanthropic plants inhabiting areas polluted with heavy metals, an advantage in interspecific competition is gained by endophytes that can live in the presence of this pollution, while also demonstrating the capacity to effectively promote plant development in areas subject to intense anthropopressure [93,141]. This is illustrated by the action of endophytes of synanthropic plants in an iron-ion-polluted environment. This element effectively and rapidly inhibits growth and negatively affects leaves and roots, so limiting its availability to plants appears crucial for their proper development. Numerous bacterial genera like *Pseudomonas*, *Azotobacter*, *Bacillus*, *Rhizobium*, *and Enterobacter* are able to produce siderophores, compounds that have a direct role in modulating the bioavailability of iron ions, by chelating iron ions and binding them to complexes, hence facilitating plant development in environments characterized by elevated iron concentrations [142,143]. Among these, *Pseudomonas* strain GRP3, *Pseudomonas fluorescens* C7, *Streptomyces* spp., *Escherichia coli*, *Arthrobacter* spp., and *Enterobacter quasihormaechei* are endophytic representatives [144,145]. As with all secondary metabolites generated by endophytes, this is essential to their survival, as due to the synthesis of siderophores, endophytic microorganisms can absorb Fe ions and use them as one of the essential minerals for their survival. In addition, they diminish the bioavailability of this element for other microorganisms that are potentially deleterious to the plant and are undeniably endophyte competitors [146]. Siderophores predominantly bind iron ions, but it has been demonstrated that they can also bind ions of other metals, including copper, gallium, cadmium, aluminum, and lead [21,133,146]. In the case of *Polygonum acuminatum*, a plant often found in Japan, several endophytic fungi were identified and isolated during research that examined the composition of endophytic microflora in settings polluted with cadmium, lead, and copper, as well as in non-contaminated situations. Significantly, the study revealed that the abundance of fungi with the ability to produce siderophores was greater in the environment subjected to the stress factor compared to conditions that were considered optimal. Moreover, *Polygonum pubescens*, serving as an exemplar of the identical genus, exhibits the capacity to thrive in environments characterized by elevated levels of cadmium, lead, and iron. This finding suggests that the plant sustains the growth of endophytic microorganisms due to their assistance in managing heavy metal ions [147]. Within the realm of endophytic bacteria, the *Rahnella* strain exhibits remarkable resilience to three specific heavy metals, namely, cadmium carbonate (CdCO_3_), lead carbonate (PbCO_3_), and zinc phosphate (Zn_3_(PO_4_)_2_). This strain, obtained from plant root tissues obtained from a polluted environment, not only demonstrates an exceptionally high tolerance to these heavy metals but also effectively solubilizes their respective compounds [148,149,150]. Additionally, the *Rahnella* strain exhibits the ability to produce siderophores, and plant phytohormones such as indole-3-acetic acid, and it can even dissolve inorganic phosphates. The investigation into the unique characteristics of this particular strain, along with its impact on enhancing the accumulation of heavy metals in plant tissues, specifically in the seedlings of rapeseed (*Brassica napus*), presents a significant area of interest for future studies in the realm of efficient phytoremediation of lead (Pb), cadmium (Cd), and zinc (Zn) ions [150,151,152,153,154].

The synthesis of phytohormones and the regulation of their synthesis by the plant host represent a prominent mechanism via which endophytes can effectively promote the growth of their host plant. Endophytic microorganisms strive to enhance the synthesis of auxins (e.g., indole-3-acetic acid (IAA)), gibberellins, or cytokinins, thus influencing the orientation of tissue development towards the root system, specifically by promoting its expansion [2]. This is accompanied by an increase in the absorptive surface, through which nutrients are absorbed and utilized not only by the plant but also by endophytes. Indole-3-acetic acid (IAA) is a phytohormone that is synthesized in significant quantities by a wide range of endophytic microbes, including both bacterial and fungal species. It is noteworthy that the action of IAA extends beyond growth stimulation [155]. The *Pseudomonas fluorescens* Sasm05 strain resides inside the tissues of *Sedum alfredii*. Hence, it exhibits the ability to accumulate zinc and cadmium from its surroundings. Additionally, this strain serves as a signaling molecule, facilitating communications between the bacterium and the host organism. Furthermore, the molecule is generated by the endophyte in response to elevated zinc concentrations in the surrounding environment. Additionally, it regulates the expression of genes that are important for the absorption and storage of this substance inside the plant [156].

From the perspective of the plant host, a significant concern is the protection against detrimental microbes, diseases, and predators, in which endophytes play a role. The host is protected against unfavorable biotic influences due to the synthesis of a wide range of secondary metabolites possessing antibacterial characteristics [74,157]. The advantages of this situation are reciprocal. By constraining or perhaps halting the proliferation of alternative bacteria, the likelihood of pathogenic germs infiltrating plant tissues, which frequently results in significant harm, is diminished. In relation to the endophytes themselves, this phenomenon offers two primary benefits. Firstly, it mitigates interspecies competition by creating a more stable and favorable environment for their survival and nutrient acquisition. Secondly, by counteracting the detrimental effects of pathogens on the host, endophytes create optimal conditions for their own growth and development. In the event of plant damage or a decline in its overall condition, the plant’s tissues would initiate induced systematic resistance (ISR) mechanisms [158,159]. These mechanisms are designed to eliminate the threat and operate in a non-specific manner, which may potentially pose a risk to endophytes. This risk can be likened to the impact of antibiotics on probiotic microflora [67].

### Genomics for Synthesis of Bioactive Compounds in Endophytic Bacteria

The complete elucidation of the biological roles of bacterial endophytes and their molecular interactions with plant hosts remains incompletely understood. Nonetheless, recent advancements in molecular biology techniques have significantly contributed to the advancement of knowledge in this field [4,24,39,160]. The involvement of endophytic genes and their expression has garnered significant attention in various areas, including the maintenance of endophytism, promotion of plant growth, biocontrol activity, and alleviation of different stresses [39,160]. Additionally, these genes have been implicated in the production of bioactive compounds [161,162,163]. The identification of these genes and a comprehensive understanding of their function, along with the examination of the genomic-level interactions between endophytes and their plant hosts, are imperative for effectively enhancing sustainable crop production. Furthermore, this knowledge is essential for the advancement of products that could potentially be utilized in the pharmaceutical and industrial sectors [137].

Advances in genomics and other “omics” techniques (metagenomics, metabolomics, transcriptomics, proteomics, etc.) have enabled a novel method for analyzing the role of gene expression in these previously mentioned traits [164]. A growing number of sequenced endophytic genomes has facilitated the exploration of several enigmatic aspects through comparative genomics investigations. The process of plant colonization by endophytic bacteria is widely recognized as a highly complex process [2]. However, recent findings have demonstrated that endophytic colonization has a significant impact on the genome of the host plant [165]. Additionally, it has been observed that genetic factors within the host plant play a crucial role in regulating the establishment of endophytes within root nodules [166]. Also, a study conducted by Monteiro et al. (2012) revealed that when closely related bacterial species were compared, they exhibited potential genes and other genetic factors associated with endophytic behavior during colonization rather than harmful activity [167]. Similarly, transcriptomics is used to investigate the endophytic microbial communities associated with other organisms and their potential role in gene expression regulation, as well as changes in gene expression patterns during exposure to potential pathogenic factors [168]. Proteomics provides information on the functional expression of the genome, primarily through mass spectrometry (MS) techniques, but it is insufficient and ineffective without genome studies [164,165].

Given the complex nature of the interactions between endophytes and their host organisms, as well as the diverse range of intermediary metabolites that are involved, analysis of the relationship should focus on the collective set of genes and their expression rather than individually [39,164]. Dudeja et al. (2021) and Pinski et al. (2019) provide comprehensive descriptions of the role of distinct genes in many aspects of endophytic bacteria–plant host interactions, including colonization, establishment of endophytism, and stress protection [39,160]. Despite the fact that previous studies have made significant progress in elucidating the genomic aspects of endophytes, it is evident that further investigation is necessary to fully comprehend this field of study.

The conventional strategy of searching for some putative properties of the given microbe strain and thus the bioactive compounds responsible for them includes cultivation- and bioassay-based screening, predicted on the basis of available literature data or knowledge about its isolation place, which may indicate its abilities (e.g., contaminated soil). Subsequently, this is followed by the purification, detection, and characterization of the compounds using chromatography in conjunction with mass spectrometry (MS) and/or nuclear magnetic resonance (NMR) techniques [169,170]. This basic method can be altered to induce gene expression and therefore produce more bioactive compounds by using the “one strain, many compounds” (OSMAC) approach (e.g., changing medium composition, cultivation conditions, co-cultivation with another strain, or adding epigenetic modifiers) [171,172]. Still, it is an untargeted method that does not give clear answers on which specific metabolites we can expect and does not exploit the strain’s potential.

Genome mining presents an alternative approach to the conventional method of secondary metabolite discovery. This strategy holds the potential to uncover all genes responsible for encoding secondary metabolites, thereby reducing reliance on serendipitous discovery [135,161,173]. Within microorganisms, including endophytes of both fungal and bacterial origin, there exists a phenomenon wherein genes responsible for the synthesis of secondary metabolites are found in close proximity to one another within the genome. Examples of such gene clusters include nonribosomal peptide synthetases (NRPSs), ribosomally synthesized and post-translationally modified peptides (RiPPs), and polyketide synthetases (PKSs) [174,175,176,177,178]. One of the primary benefits of an organized operonic structure, which is extensively dispersed across the genome with approximately 30–50% of all genes being grouped in this manner, is the presence of a single promoter that governs the regulation of gene expression, leading to co-transcription [179].

The development of whole-genome sequencing (WGS) techniques facilitated the initial exploration of genome mining in *Streptomyces coelicolor* and *Streptomyces avermitilis*, two extensively studied species that were previously considered to have been fully utilized. These investigations unveiled the untapped capabilities of these organisms [173,180,181,182,183]. Subsequently, there has been substantial progress in bioinformatic methodologies and, therefore, genome mining, leading to the identification of numerous novel bioactive compounds derived from diverse endophytic bacteria [184,185]. This includes the isolation of compounds from herbaceous plants, such as those documented in Table 2. In a recent study conducted by Tsalgatidou et al. (2022), a wide range of genes encoding antimicrobial compounds were identified in *Bacillus halotolerans* from *Calendula officinalis*. These compounds included lipopeptides such as fengycin, surfacin, and mojavensin A, as well as siderophores such as bacillibactin [186]. This discovery builds upon previous research by Jasim et al. (2016), who also found various lipopeptide genes for surfactin, iturin, and fengycin in endophytic *Bacillus* sp. from *Bacopa* [187]. The process of genome mining encompasses more than just the discovery of genes and/or biosynthetic gene clusters (BGCs). It also involves the analysis of biosynthetic pathways for chemicals and the prediction of their functional and structural characteristics [125,126]. The approach relies on the utilization of synthetic biology and bioinformatic tools, including but not limited to antiSMASH [126,127], RODEO [128], and ThioFinder [188], among others. The ease of comparing collected data with database-presented biosynthetic gene clusters (BGCs) and identifying homologs has been facilitated by the accessibility of DNA sequences and other genomic data uploaded in public databases [125,189]. It has been observed that BGCs are predominantly inactive or exhibit low expression levels during standard laboratory cultivation, necessitating their activation for gene expression [161,190,191]. The various methods for awakening microbial cells from a dormant state encompass heterologous expression, ribosome engineering, co-cultivation, optimization of regulatory networks (such as employing more potent promoters), and the introduction of chemical elicitors. The activation mechanisms of silent (or cryptic) BGCs have been extensively examined in a comprehensive study by Rutledge [191]. Additionally, the utilization of mutasynthesis and/or genetic engineering, commonly referred to as combinatorial biosynthesis, may serve as a crucial measure in this process and offer insights into the pathways involved. By creating mutant microorganisms with inactivated or deleted biosynthetic gene clusters (BGCs) or specific individual genes, the production of certain compounds can be eliminated [125,192]. Ultimately, the process of purifying and isolating newly discovered bioactive compounds is executed, resulting in molecules that are now prepared for subsequent activity assessments and structural analysis.

One notable advantage of genome mining is its ease of application in laboratory settings, primarily due to its cost-effectiveness and lack of a need for specialized skills required by researchers. Additionally, genome mining offers the opportunity to explore novel compounds, as conventional methods often result in a high rate of rediscovery. However, it is important to acknowledge the limitations of this approach, including its ability to only identify previously synthesized BGCs or similar ones, as well as its inability to predict the biological activities of the identified compounds [173,180,181]. Encouragingly, there is a notable expansion in databases and significant advancements in synthetic biology, which have the potential to effectively address these challenges in coming years.

## 7. Ruderal Plants

For centuries, mankind has co-existed with synanthropic plants. They occur in locations where humans have either mistakenly or consciously destroyed the native plant cover, changing nature for their own benefit. Environmental factors in synanthropic settings necessitate specialized plant adaptations. These include reducing their growth cycle so that they can generate many generations in a single growing season, increasing the number of seeds produced, having a high ability for vegetative reproduction and competitiveness, and having broad adaptability, including adaptations to plant protection chemical compounds.

Synanthropic plants are divided into segetal and ruderal species. Segetal plants are typically weeds that grow alongside field and garden crops (from Latin *seges*—sowing, cultivated field) [193]. 

The term ruderal comes from the Latin word *rudus* and describes species adapted to disorders [194]. As a result of human activity, ruderal areas are constantly fed with nutrients and thus are characterized by the presence of highly productive flora and a variety of competitive relationships associated with dynamic, continuous, or occasional interference and hasty successive changes [146]. Ruderal plants include species from environments that are directly or indirectly influenced by humans and that can grow in disturbed artificial habitats, such as home surroundings, gardens, roadsides, railroads, etc. [10,195]. Additionally, they are frequently observed in semi-natural environments such as disturbed river margins and forest edges [193,196,197]. They flourish there despite human intervention. Because these ecosystems have a high concentration of mineral salts, the ruderal vegetation is mostly nitrophilous (nitrogen-loving) plants. Their communities are quite dynamic; their composition changes as they grow, and they occasionally initiate secondary succession [198].

Ruderal plants are resistant to competition as they easily adapt to new environments and rapidly form large quantities of seeds that can be dispersed even by car tires. That is why these plants are also known as pioneer plants. A common feature of most ruderal species is their ability to foster beneficial microorganisms around their roots, which, in turn, helps them cope better with environmental stress [194]. Moreover, their seeds require a small amount of nutrients for germination, their roots develop quickly, and they can form mycorrhizae [195]. 

### 7.1. Ruderal Herbaceous Plants

Ruderal plants are commonly referred to as pioneer plants because of their ability to easily adapt to new conditions and colonize new areas. The predominant species within this group include small annual herbaceous plants. These plants are a great source of biologically active compounds, and their use in medicine has been known for centuries [199].

The global market for plant-based products is estimated at USD 83 billion and continues to grow. According to Wangchuk, (2018), about 85% to 90% of the worldwide population relies on traditional medicine [200,201]. Nowadays, in less developed countries, more than 3.3 billion people utilize medicinal plants regularly. According to the World Health Organization (WHO), traditional medicinal plants are defined as natural resources that can be used in industrial processing for the treatment of diseases on a local or regional scale. It is estimated that between 750 thousand and 1 million different plant species grow in the world, where around 500 thousand have been identified and named. According to WHO, the number of plants used for the treatment is around 20 thousand. Traditional medicine and plant species are generally used by patients who have chronic medical conditions such as cancers (2%), liver diseases (21%), HIV (22%), asthma (24%), and rheumatologic disorders (26%) [202].

Herbaceous plants are an abundant source of biologically active compounds. Their usage is growing, as they serve as raw materials for the extraction of active ingredients that are later used in the synthesis of various drugs. Relevant laxatives, blood thinners, antibiotics, and antimalarial compounds are ingredients of many drugs, and they are mainly obtained from plants. Taxol, vincristine, and morphine isolated from foxglove, periwinkle or yew, and opium poppy, respectively, are examples of such compounds [203,204]. 

### 7.2. Biotechnological Potential of Endophytic Bacteria from Ruderal Herbaceous Plants

Ruderal plants experience a range of biotic and abiotic challenges throughout their lifespan because of their growing environment. These stresses include exposure to several xenobiotics, such as herbicides, pesticides, hydrocarbons, and heavy metals. Nevertheless, they manage to endure in such a hostile habitat due to the presence of microbes residing within their tissues [46].

The presence of endophytic microbes in plants provides several advantages, such as enhanced production and fitness, improved stress tolerance, and better resistance to diseases. Thus, their reliance on microbes that produce secondary metabolites is evidently crucial to their long-term survival strategy [10].

#### 7.2.1. *Chelidonium majus* L.

*Chelidonium majus* L. is a ruderal plant indigenous to Europe, Asia, and South America. The plant, despite its toxicity, generates numerous bioactive compounds such as alkaloids that possess potent antibacterial, antiviral, and even anticancer properties [111,113,205]. Therefore, it is essential to isolate endophytes from the plant and examine the possibility of sharing the ability to produce the same biologically active compounds. Goryluk et al. (2009) isolated 34 endophytic bacteria from internal stem tissues of Chelidonium majus L. and investigated their antifungal properties against six fungal species, namely, *Alternaria alternata*, *Paecilomyces variotti*, *Aureobasidium pullulans*, *Byssochla-mysfulva*, *Chaetomium* sp., and *Exophiala mesophila*. The authors observed that eleven isolates demonstrated the suppression of fungal growth, with the exception of *B. fulva*. A single bacterial strain demonstrated broad-spectrum antifungal activity against all the fungi examined. The strain was categorized as *B. amyloliquefaciens* based on the API-20E, -50CHB tests, and the study of the 16S rDNA sequence [113]. Studies on endophytic microorganisms from *Chelidonium majus* L. were extended by Marchut-Mikolajczyk et al. (2018) [111]. The authors isolated 11 endophytic bacteria from the tissues of *Chelidonium majus L*. plants growing in a motorway neighborhood. The researchers investigated the capacity of isolated microorganisms to degrade hydrocarbons (namely, diesel oil and waste motor oil) and produce biosurfactants. Every strain that was examined exhibited degrading activity. Nevertheless, strain 2A exhibited the most pronounced degrading activity towards both diesel and waste engine oil. The strain also demonstrated the greatest biosurfactant production. The strain was categorized as *Bacillus pumilus* based on the study of the 16S rDNA sequencing. The authors assert that the biosurfactants created not only improve the breakdown of hydrophobic substances but also have the potential to stimulate plant development in a polluted environment [111]. While the data presented are novel and captivating, the utilization of the strain and biosurfactant can only occur once a comprehensive understanding of the underlying mechanism of the observed phenomenon is obtained. This knowledge is crucial in assessing the feasibility of applying biosurfactants to enhance plant growth, particularly in polluted regions.

#### 7.2.2. *Urtica dioica* L.

*Urtica dioica* L. (stinging nettle) is a common plant found in northern Europe and much of Asia, primarily in rural areas. The beneficial impact of *Urtica dioica* on human well-being has been globally recognized since ancient times. It is utilized for the treatment of several ailments and conditions such as hemorrhoids, eczema, rheumatism, bronchitis, hyperthyroidism, and cancer. Importantly, it is noteworthy that this treatment does not have any adverse effects [206]. Thus, it is highly probable that endophytic microorganisms possessing unique capabilities reside within the internal tissues of stinging nettle [146]. 

Naoufal et al. (2018) successfully obtained 54 endophytic bacterial strains from *Urtica dioica* L. [207]. From this collection, the authors chose Gram-positive isolates belonging to the Bacilli genera that exhibited distinct morphologies and capabilities. The primary objective of this research endeavor was to identify the most effective phytopathogen inhibitors. The authors identified three isolates that demonstrated the most potent antagonistic activity against common phytopathogens (*Rhizoctonia solani*, *Fusarium oxysporum*, *Phytophthora parasitica*, and *Colletotrichum gloeosporioides*) based on biochemical analysis. The average value of phytopathogen growth inhibition was 73.21% when compared to the control sample, which lacked endophytic bacteria. Additionally, the authors documented that these species of endophytic bacteria might serve as a reliable reservoir of various enzymes and secondary metabolites [207]. 

Krimi et al. (2016) assessed the capacity of endophytic bacteria from four weeds (including stinging nettle) to inhibit the development of tomato plants and function as biocontrol agents against bacterial phytopathogens [107]. The seventy-three bacterial isolates isolated by the authors, each possessing eight distinct morphological profiles, exhibited antagonistic activity against a minimum of two pathogenic bacteria out of the seven that were tested [107]. Three isolates of *Bacillus amyloliquefaciens* OR2, *Bacillus pumilus* OS2, and *Bacillus methylothrophicus* OS4 were isolated from *Urtica dioica* L. The growth of *Agrobacterium vitis*, *Clavibacter michiganensis* subsp. *michiganesis*, and *Xanthomonas axonopodis* was significantly inhibited by 20–30.3 mm when exposed to the OR2 and OS2 strains. The growth of a greater variety of phytopathogens was significantly inhibited by the OS4 strain, including *Agrobacterium tumefaciens* (40 mm), A. vitis (34.6 mm), C. michiganensis subsp. michiganensis (40.3 mm), Pectobacerium spp. (33 mm), and *Ralstonia solanacearum* (41.3 mm). Moreover, Krimi et al. (2016) documented that endophytic strains derived from *U. dioica* L. exhibited significant growth-promoting activity on tomatoes, with the OS4 strain in particular significantly accelerating their development under in vitro and in vivo conditions. This acceleration was attributed to the endophytic strains’ secretion of hormones including indole-3-acetic acid (IAA) and ethylene [107]. The potential of *U. dioica* endophytic mircoorganisms in biofertilizer and biopesticide formulation has been suggested by their capabilities [107]. Furthermore, Marchut-Mikolajczyk et al. (2023) successfully isolated nine strains of endophytic bacteria from common nettle [146]. Among these, three strains were chosen based on their capacity to biosynthesize polyphenols. The identified isolates comprised one strain of Bacillus mycoides and two strains of *Bacillus cereus* [146].

#### 7.2.3. *Plantago lanceolata* L.

*Plantago lanceolata* L., also known as ribwort plantain, is a highly prevalent weed found in Europe, Asia, the Americas, and Australia, where it has been introduced as a non-native species. Traditionally, it was employed as a cure for wound healing in folk medicine. However, it also exhibits anti-inflammatory, analgesic, analeptic, antihistaminic, and other actions [208]. Plantains are often seen growing along roadsides and other polluted locations; thus, they might potentially be used as a bioindicator for the buildup of heavy metals in soil. However, our understanding of the bacteria residing within their tissues remains limited. Tello et al. (2014) reported the presence of the fungal endophyte species *Hygrocybe virginea* in the roots of *Plantago lanceolata* and provided evidence that it may be systemic endophyte transmitted with the plant seeds (vertically) [209]. In addition, Krimi et al. (2016) successfully isolated five endophytic bacterial strains from the roots. Among these strains, two were recognized as *Bacillus methylotropicus* and *Pseudomonas brassicacearum*, while the remaining strains belonged to the *Bacillus* spp. Genus [107]. These two strains have demonstrated the highest capacity as biocontrol agents against *Agrobacterium* spp. and *Pectobacterium* spp., as well as other isolates [107].

#### 7.2.4. *Matricaria chamomilla* L.

*Matricaria chamomilla* L. (chamomile) is another widespread herbaceous plant indigenous to Europe and is broadly used in traditional medicine in the form of herbal tea, mostly for gastrointestinal issues. Essential oils derived from fresh or dried flower heads are currently used in a great number of cosmetics, perfumes, baked goods, and beverages. The potential benefits and bioactive properties of chamomile have been extensively studied over the years [208,210,211,212]. However, only a few endophytes have been isolated from its tissues to this day. Studies conducted by Köberl et al. (2013) identified endophytes from the inner root tissue of *Matricaria chamomilla* as *Paenibacillus polymexa* strain Mc5Re-14, and so far, this is the only endophytic bacteria from this plant that has been characterized and sequenced [115,213]. Its genome encodes many synthases (including a few polyketides), chitinase, extracellular glucanases, and genes responsible for auxin and spermidine production, which is reflected in its activity against phytopathogenic fungi (*Verticillium* sp., *Fusiarium culmorum*, *Rhizoctonia solani*) and the human pathogen *Escherichia coli*. In the work of Erjaee et al. (2019), chamomile was one of the plants that had the highest number of isolated bacterial endophytes compared to the studied medicinal plants. Two out of sixteen isolates exhibited antifungal activity for all five tested food spoilage fungi species [99]. Another interesting study conducted on *Marticaria chamomilla* by Schmidt et al. (2014) investigated the impact of various bacterial inoculants on the microbiome structure of the plant and the production of secondary metabolites. Pyrosequencing analysis of the 16S RNA gene libraries showed significant differences in the bacterial diversity between the treatments, and for two inoculating Gram-positive species—*B. subtilis* Co1–6 and the previously mentioned *P. polymyxa* Mc5Re-14—higher yields of apigenin-7-O-glucoside were also obtained in comparison to treatment with Gram-negative strains [214]. This indicates the importance of understanding the influence of bacterial inoculants and the risks of using them as biocontrol agents, which should be examined in-depth in further research.

#### 7.2.5. *Equisetum arvense* L.

*Equisetum arvense* (field horsetail) is a plant common throughout Europe and the rest of the world, where it is found in a wide range of habitats, colonizing ruderal and segregated areas in abundance [208]. A characteristic feature of its tissues is a high content of silica and other compounds such as alkaloids, saponins, and phenolic compounds, which directly translates into the use of this plant as an herbal resource [211,212]. Infusions of the above-ground parts of the herb are a source of easily absorbed silica that has a particular effect on the circulatory and urinary systems. The herb itself also exhibits a diuretic effect, thus preventing excessive accumulation of this compound in the body. Horsetail is also used for things like diabetes and edema and for the general improvement of hair and nails [211].

*E*. *arvense* was analyzed for its endophytes, and the number of isolated bacteria showed how great their contribution to the colonization of this plant is. In their study, Das et al. (2017) obtained 103 bacterial strains, most of which were identified based on morphological characteristics and using 16S rDNA molecular techniques [116]. Ten bacteria showed promising anticandidial activity, and the bacteria of greatest importance appeared to be *Psychrobacillus insolitus,* generating the most extensive zones of inhibition for *Candida albicans* and *Curtobacterium oceanosedimentum* and effectively inhibiting the growth of *Candida glabrata*. Butanol metabolite extracts of these bacteria caused lysis of the cell membrane, resulting in fungal cell death [116]. Its antimicrobial activity has also been analyzed against common foodborne pathogens, specifically *Staphylococcus aureus* and *Escherichia coli* O157:H7. Three strains—*Streptomyces albolongus*, *Dermacoccus* sp., and *Mycobacterium* sp.—proved to be effective against S. aureus, while for the analyzed *E. coli* strain, the best growth inhibitors were *Streptomyces griseoaurantiacus* (EAL196) and *Paenibacillus* sp. (EAS116), whose results, however, were only moderate. The cell death of the pathogenic bacteria was most likely caused by the penetration of endophytic metabolites into their cells, which caused the disruption of various metabolic functions [215]. In their study, Woźniak et al. (2019) successfully isolated a total of twenty-three endophytic bacteria from six distinct plant species, one of which was *Equisetum arvense* L. Among these bacteria, they identified five unique endophytic strains [117]. The researchers investigated the process of phosphate solubilization, the synthesis of IAA-like compounds and siderophores, as well as nitrogen fixation. Additionally, they analyzed the phenotypic characteristics of the strains. The majority of the endophytic bacteria strains exhibited increased production of indole-3-acetic acid (IAA) at levels over 10 μg IAA/mL, with *Stenotrophomonas maltophilia* ES2 displaying one of the highest levels. Additionally, the strains were analyzed for their ability to produce siderophores. *Comamonas koreensis* ER1 was shown to be one of the most prolific producers of siderophores. The effectiveness of N2 binding by bacterial strains was also assessed. After being incubated in a bacterial culture medium for 72 h, the effectiveness of nitrogen fixation in *Rhizobium* sp. ES1 rose by a factor of 1.67. The findings indicated that the application of *Equisetum arvense* L. endophytes has the potential to enhance plant development [117]. The relatively high anticandidal and antimicrobial activity may be the start of new research to develop natural agents for both combating candidiasis and compounds for use in the food industry. *Equisetum arvense* L. still represents a reservoir of many potential secondary metabolites of endophytes and, in this regard, requires further research [116,117].

#### 7.2.6. *Oenothera biennis* L.

*Oenothera biennis* (biennial evening primrose) is a native plant of South America and is common in Europe. The oil extracted from the seeds is used for medicinal purposes, as it lowers blood pressure and cholesterol and affects blood sugar levels positively. It serves as the best studied species among its family, and studies are underway to learn more about its chemical composition and biological activity, including studies of endophytes [208].

As it is a plant that readily grows in sandy, loose substrates and has a relatively high resistance to pollution (including hydrocarbon pollution), it is particularly keen to colonize industrial neighborhoods and railroad areas. This suggests a link to endophytes increasing tolerance to agents such as petroleum hydrocarbons. This aspect was investigated by Pawlik and others (2017), proving that the majority of endophytic bacteria colonizing evening primrose tissues were representatives of *Gammaproteobacteria* as well as *Alphaproteobacteria* and *Actinobacteria*. Species from the genera *Rhizobium*, *Rhodococcus*, and *Xanthomonas* were identified. Of the bacterial isolates, more than 90% were capable of utilizing diesel fuel as a carbon source, equally often kerosene oil, while some of them (about 30%, including all the *Rhodococcus* strains) also utilized n-hexadecane. As in other cases, the diversity of the endophytic flora in the stressed environments was relatively low [119].

Regarding the endophytic mechanisms promoting plant growth, many of the bacterial strains isolated from *O. biennis* tissues were capable of synthesizing IAA and hydrogen cyanide (*Stenotrophomonas* sp), and some were also siderophores (*Pseudomonas umsongensis*). Around half of the strains possessed the acdS gene in their genome, which enabled them to produce the enzyme ACC (1-aminocyclopropane-1-carboxylic acid) deaminase. This enzyme utilizes the precursor of ethylene to reduce its concentration in plants, thereby mitigating its negative impact on plant growth. Additionally, as *O. biennis* is a synanthropic plant, this ability to decrease ethylene levels also enhances its spread [119].

#### 7.2.7. *Silybum marianum* L.

*Silybum marianum* L. (commonly known as milk thistle) is a highly demanded herb growing in central Europe; however, it originally grew in the Mediterranean basin, long known for its hepatoprotective effect on the liver. This is due to its extract—silymarin—which is a unique mixture of seven flavonolignans (silybin A and B, isosilybin A and B, silychristin, isosilychristin, and silydianin) and a flavonoid (taxifolin) [208,212]. Silymarin complexed with phosphatidylcholine and glycol conjugates for better absorption and delivery is safe for broad therapeutic use with minor side effects and no life-threatening adverse events [216,217,218]. An isolation of endophytic bacteria was conducted by Anwar et al. (2023). Anwar’s team isolated a total of eleven endophytic bacteria from *S. marianum* plants that were harvested from locations contaminated with heavy metals. These bacteria were identified using 16s rRNA sequencing. Three isolates exhibited several characteristics, such as robust resistance to heavy metals, promotion of plant development, regulation of plant hormones, remediation of heavy metal toxicity, and antibacterial activity. The isolates SJLC and SJRB exhibited the highest production of indole-3-acetic acid (IAA). These isolates were recognized as *Bacillus* sp. and *Lysinibacillus* sp., respectively. The production of IAA led to an improvement in both root and shoot length. The SJLC isolate showed efficacy against four of the investigated infections. [147,219]. 

#### 7.2.8. *Mentha piperita* L.

*Mentha piperita* (peppermint) is a naturally occurring hybrid, and although it is a plant native to the Mediterranean region, it is now settled and cultivated all over the globe. It is also abundant in European territories; in Poland, it is the most common species of mint. It is used in various industries, finding application in food and pharmaceuticals, among others. Peppermint essential oil has become the most widely used product of this type precisely because of its taste and a number of properties, such as antimicrobial activity. However, thanks to Juyal et al.’s (2017) research, we know that the substances found in the essential oil itself are not only responsible for this particular activity (e.g., menthol) but also the endophytes present in their tissues and the secondary metabolites they produce [220]. At this point, there is no evidence that *M. piperita* endophytes increase menthol levels as they synthesize it in *Picrorhiza kurroa* Royle ex Benth. The endophytic solvent fractions of the predominant strains among the bacterial endophytes inhabiting mainly the underground parts of *M. piperita* have been shown to have strong antimicrobial activity against selected strains of *Bacillus*, *Micrococcus*, and *Pseudomonas* [220] 

Shokhiddinova and Normurodova conducted research on the production of enzymes by endophytes of medicinal plants, including *Mentha piperita* L. They found that all of the endophytic bacterial isolates (14 strains) extracted from the roots, stems, and leaves had affinity for 1% casein, which indicates protease production [221]. 

## 8. Future Perspectives

Synanthropic plants are widespread and can be found in almost every region of the globe. These organisms possess the capacity to withstand adverse environmental circumstances, including high salinity, drought, reduced light exposure, and the presence of toxic substances, such as heavy metals, hydrocarbons, or other pollutants, due to their ability to adapt to the escalating anthropogenic pressure they experience. These features and abilities are acquired via the process of adaptation. Consequently, this makes them a good place to find endophytic microorganisms that exhibit distinctive properties. Fungal endophytes have been described in several scientific articles as having the ability to produce physiologically active chemicals such as phytohormones, surfactants, immunosuppressants, anticancer agents, and antibiotics. Nevertheless, research on bacterial endophytes is still in its infancy, especially when it comes to synanthropic plants. These, in contrast to their non-anthropopressured plant relatives, may either generate more of the same physiologically active compounds or entirely new ones.

This suggests that these bacteria and the novel bioactive compounds they generate may have far-reaching effects in many fields, both now and in the future. These fields include the agriculture, pharmaceuticals, medicine, food, chemicals, tanning, and medical fields.

A comprehensive bioprospecting investigation of endophytic microorganisms from various ecological habitats, such as extreme environments (ruderal, salted, dry, hot, cold, devastated) and the marine environment, is essential for the identification and characterization of unique endophytes possessing specific attributes that could prove valuable in diverse applications within the agricultural, chemical, and medicinal industries. In the near future, there will likely be a shift in practice towards emphasizing the optimization of the interaction between plants and soil microorganisms and endophytes. The molecular pathways that govern the connection between plants and endophytes have not yet been discovered. They will offer a fresh avenue for the isolation and characterization of innovative compounds for human consumption as well as a revolutionary approach to enhancing agricultural productivity and environmental sustainability.

## 9. Conclusions

Synanthropic plants, which have successfully acclimated to harsh climatic conditions, serve as a valuable reservoir of endophytes. These microorganisms have the ability to generate distinct bioactive substances that could be extensively utilized in diverse sectors such as the agriculture, pharmaceutical, medicine, and the chemical industries. Nevertheless, there remains an immense number of plants yet to be investigated in the search for microorganisms possessing unique characteristics. The identification of novel endophytic species obtained from a plant thriving in harsh conditions is a chance to discover microbes, representing a promising opportunity for various biotechnological uses, namely, exhibiting strong metabolic activity against various pollutants, enhancing agricultural productivity, creating novel pharmaceuticals, and generating unique chemical substances. The continued investigation and advancement of genetic analysis techniques are essential in order to fully exploit this potential.

## Figures and Tables

**Table 1:** The most frequent and widely recognized methods of plant material surface sterilization [52].

No.	Method of Plant Material Sterilization	Plant or Plants Part Example	Endophytes Isolated	Reference
Single compound approach				
1	EtOH 70–99% (different times)	*Nepenthes species; Anemone nemorosa*, *Ranunculus ficaria*, *Vaccinium oxycoccus*, *Sambucus nigra*	*Acremonium* sp. *Libertella heveae*	[53,54,55]
2	NaOCl 2–30% (up to 30 min)	leaves stems and roots *Pistacia atlantica* L.; *Simmondsia chinensis*	*Total of 61 endophytes*, *with 10 belonging* *to genus Pseudomonas*, *Stenotrophomonas*, *Bacillus*, *Pantoea* *and Serratia; Bacillus* sp., *Methylobacterium* *aminovorans*, *Oceanobacillus kimchi*, *Rhodococcus pyridinivorans*, *and* *Streptomyces* sp.	[56,57]
3	0.1% HgCl_2_ (1–10 min)	*Ziziphus nummularia*	*Microsporum gypseum*, *Aspergillus fumigatus*, *Aspergillus calidoustus*, *Penicillium viridicatum*, *Trichophyton tonsurans*, *and Penicillium marneffei*	[58]
4	Surfactants	Leaves, stems, and roots		[59]
5	Tween 20 0.01–10%	Leaves, stems, and roots	127 Endophytic fungi	[60]
6	Tween 80 0.1%	Fruits		[61]
7	Teepol 5%	Leaves, stems, and roots	228 Isolates representing at least 19 genera of actinobacteria	[62]
8	Triton X-100 0.05–0.1%	Leaves, stems, and roots	116 endophytic fungi	[63,64,65]
10	Hydrogen peroxide 3–90%	Leaves, stems, and roots		[59,66]
Combined approach				
11	2% NaOCl + 0.1% Tween 20	Roots (tomato)	*Rhizobium*, *Bacillus*, *Microbacterium*, *Enterobacter species*	[67,68]
12	70% EtOH-3.125% NaOCl	*Psidium guajava and Ziziphus mauritiana*	7 Endophytic actinomycetes	[53]
13	75% EtOH (2 min) and 1.5%NaOCl (3 min)	*Brassica olerocea*, *B. rapa*, *and Raphanus sativus*	4178 Endophytic fungal isolates belonging to 51 different genera	[69]
14	70% EtOH (1 min), 2.5% NaOCl (3 min); 70% EtOH (1 min), 2.5% NaOCl (1 min), and 70% EtOH (30 s)	Roots and leaves of *Salvia abrotanoides*	56 Endophytes	[70]
15	75% EtOH (2 min), 2% NaOCl (3 min), and 75% EtOH (30 s)	*Gnetum gnemon*	*Staphylococcus warneri*, *Solibacillus* *isronensis*, *Bacillus megaterium*, *Caballeronia glebae; Bacillus* *licheniformis*, *Bacillus velezensis*, *and* *Bacillus atrophaeus*	[71]
16	75% EtOH (1 min), 1% HgCl_2_ (4 min), and 75% EtOH (1 min)	*Lycoris radiata*	Total of 188 bacterial endophytes	[72]
17	70% EtOH (1 min), 2.5% NaOCl (2 min), and 70% EtOH (1 min)	Stems, leaves, and capsules *of Spiranthes spiralis*, *Serapiasvomeracea, and Neottia* *ovata*	*50 bacteria belonging to genera* *Sphingomonas*, *Microbacterium*, *Pantoea*, *Staphylococcus*, *Pseudomonas*, *Bacillus*, *and Streptomyces*	[73]
18	75% EtOH (1 min), 1% NaOCl (3 min), and 75% EtOH (30 s)	*Artemisia nilagirica*	*Arthrobacter* sp., *Bacillus* sp., *Burkholderia* sp., *Pseudomonas* sp., *Psychrobacter* sp., *Serratia* sp., *Microbacterium* sp., *Enterobacter* sp., *Chromobacterium violaceum*, *and* *Kosakonia cowanii*	[74]
19	75% EtOH (1 min) and 5% NaOCl (8 min)	*Lycium ruthenicum*	109 Endophytic bacteria with 36 genera	[65,68]
20	Cycloheximide (100 mg/L) and nalidixic acid (20 mg/L) (1 min), 5% NaOCl 5 min), 2.5% Na_2_S_2_O_3_ (10 min), and 10% NaHCO_3_ (10 min)	*Glycine max*	70 Endophytic actinobacteria belonging to 14 genera	[75]
21	70% EtOH (3 min), 8% NaOCl (4–5 min), 2.5% Na_2_S_2_O_3_ (10 min), and 70% EtOH (1 min)	*Camellia sinensis*	46 Actinobacteria belonging to families *Streptomycetaceae*, *Thermomonosporaceae*, *Camellia sinensis*, *Nocardioidaceae*, *Microbacteriaceae*, *Dermatophilaceae*, *Nocardiopsaceae*, *Nocardiaceae*, *Mycobacteriaceae*, *Dermacoccaceae*, *Micromonosporaceae,* and *Pseudonocardiaceae*	[76]
22	70% EtOH (1 min), 1.2% NaOCl (15 min), and 75% EtOH (30 s)	*Oryza sativa*	87 Endophytic bacteria	[77]
23	2.5% NaOCl (2 min), and 75% EtOH (30 s)	*Pinus cotorta*	*77 Endophytic diazotrophs Pseudomonas*, *Bacillus*, *Paenibacillus*, and *Rhizobium*	[78]
24	Surfactant-75% EtOH (10 min), 5.25% NaOCl (10 min), and 70% EtOH (2 min)	*Phoenix dactylifera* L.	14 Endophytic bacteria	[79]
25	5% NaOCl (4–10 min), 2.5% Na_2_S_2_O_3_ (10 min), and 75% EtOH and 10% NaHCO_3_ (10 min)	12 Forest trees	2174 Actinobacteria	[80]
26	0.1% Tween 20 and 70% EtOH (3 min), 0.4% NaOCl (1 min), and 70% EtOH (2 min)	7 Medicinal plants	13 *Streptomyces* sp., *2 Microbacterium* sp., *1 Leifsonia xyli*, *1 Brevibacterium* sp., *1 Actinomycete*	[81]
30	0.01% Tween 20 (1 min), 2.5% NaOCl (2.5 min), 2.5% Na_2_S_2_O_3_ (5 min), 75% EtOH (2.5 min), and 10% NaHCO_3_ (5 min)	*Dioscorea zingiberensis*	123 Strains of endophytic fungi, most abundant genera	[77]

**Table 2:** Bioactive compounds produced by endophytic bacteria isolated from synanthropic herbaceous plants occurring in Europe.

No	Host Plant	Endophytic Bacteria	Compound/-s	Activity	Reference
1	*Achillea fragrantissima*	*Streptomyces* sp., *Nocardioides* sp., *Kitasatosporia* sp., *Kibdelosporangium* sp.	chitinase, siderophores	enzymatical, antifungal	[98]
2	*Achillea millefolium (yarrow)*	*Bacillus safensis*	volatile metabolites (butanal, 3-methyl-, 2-heptanone, 6-methyl-5-methylene-, hydrogen azide, propene, 2-butene, 6-oxabicyclo3.1.0 hexane)	antifungal	[99]
3	*Alkanna tinctoria* (dyer’s alkanet)	*Pseudomonas* sp., *Bacillus* sp.	IAA, ACC deaminase, siderophore; pectinase, ligninase	plant growth promotion; enzymatical	[100]
4	*Allium fistulosum* (spring onion)	*Streptomyces* sp. TP-AO569	fistupyrone	spore germination inhibition	[101]
5	*Allium tuberosum* (garlic chives)	*Streptomyces* sp. TP-A0595	6-prenylidole	antifungal	[102]
6	*Arctium lappa* (greater burdock)	*Bacillus* sp., *Pantoea* sp., *Microbacterium* sp., *Pseudomonas* sp.	IAA, ACC deaminase, siderophore, hydrolase	plant growth promotion	[103]
7	*Armoracia rusticana* (horseradish)	*Serratia ficaria*	siderophores, lipase, protease, chirinase	biocontrol, enzymatic, plant growth promotion	[104]
8	*Arnica montana* (mountain arnica)	*Streptomyces* sp.	glutarimide antibiotics (cycloheximide, actiphenol), diketopiperazines (cyclo-prolyl-valyl, cyclo-prolyl-isoleucyl, cyclo-prolyl-leucyl, cyclo-prolyl-phenylalanyl)	antibiotic, cytotoxic	[105]
9	*Artemisia vulgaris* (common mugworts)	*Bacillus pumilus*, *Bacillus safensis*	volatile metabolites (butanal, 3-methyl-, 2-heptanone, 6-methyl-5-methylene-, hydrogen azide, propene, 2-butene, 6-oxabicyclohexane)	antifungal	[99]
10	*Atropa belladonna* (deadly nightshade)	*Streptomyces* sp. AB100	piperazic-acid-containing peptides	antibiotic	[106]
11	*Calendula arvensis* (field marigold)	*Pseudomonas brassicacearum*	-	biocontrol, plant growth promotion	[107]
12	*Calendula officinalis* (medicinal calendula)	*Pseudomonas putida*	HCN, siderophores, lipase, protease, chitinase	biocontrol, enzymatic, plant growth promotion	[104]
13	*Matricaria* *chamomilla* L., *Calendula officinalis* L., and *Solanum distichum* Schumach. and Thonn	*Bacillus subtilis* subsp. *subtilis*, *Bacillus subtilis* subsp. *spizizenii*	-	antibacterial, antifungal, nematicidal	[108]
14	*Iris pseudacorus* L.	*Pseudomonas rhizosphaereae* FST5	IAA, ACC deaminase, HCN, chitinase, protease, glucanase, lipase	antifungal, plant growth promotion	[109]
15	*Chelidonium majus* (greater celandine)	*Bacillus velezensis*	IAA, siderophores, iturin B, iturin D, fengycin, surfactin, cellulase	biocontrol, plant growth promotion	[110]
16	*Chelidonium majus* (greater celandine)	*Bacillus pumilus* 2A	glycolipid	emulsyfing (biosurfactant)	[111]
17	*Chelidonium majus* (greater celandine)	*Bacillus amyloliquefaciens* 30B, *Erwinia persicinia* 2–5b	IAA	plant growth promotion	[112]
18	*Echinacea purpurea* (purple coneflower)	*Bacillus thuringensis*, *Bacillus amyloliquefaciens*	-	antifungal	[113]
19	*Echinacea purpurea* (purple coneflower)	*Rheinheimera* sp. EpRS3	lipases, phospholipase, protease	antibacterial, antimicrobial	[114]
20	*Elymus repens* (*couch grass*)	*Pseudomonas azotoformans*	IAA	plant growth promotion	[115]
21	*Equisetum arvense* (common horsetail)	*Psychrobacillus insolitus*, *Curtobacterium oceanosedimentum*	-	anticandidal	[116]
22	*Euphorbia helioscopia* (sun spurge)	*Comamonas koreensis*, *Stenotrophomonas maltophilia*, *Rhizobium* sp., *Brevundimonas* sp.	IAA-like compounds, siderophores	plant growth promotion	[117]
23	*Euphorbia helioscopia* (sun spurge)	*Bacillus cereus*, *Bacillus amyloliquefaciens*	-	biocontrol, plant growth promotion	[107]
24	*Euphorbia peplus* (petty spurge)	*Pseudomonas brassicacearum*	-	biocontrol, plant growth promotion	[107]
25	*Foeniculum vulgare* (fennel)	*Klebsiella pneumoniae*	siderophores, lipase, protease, chitinase	biocontrol, enzymatic, plant growth promotion	[104]
26	*Hypericum perforatum* (St. John’s wort)	*Achromobacter* sp., *Erwinia persicina*, *Stenotrophomonas* sp.	IAA, HCN, cellulase, protease, beta-1,3-glucanase	biocontrol, plant growth promotion, antifungal	[118]
27	*Iris pseudacours* (pale yellow iris)	*Pseudomonas azotoformans*	HCN, siderophores, lipase, protease	biocontrol, enzymatic, plant growth promotion	[104]
28	*Matricaria* *chamomilla* L., *Calendula officinalis* L., and *Solanum distichum* Schumach. and Thonn	*Pseudomonas gessardii* HRT18	IAA, HCN, siderophores, lipase, protease, chitinase, glucanase	biocontrol, enzymatic, plant growth promotion	[109]
29	*Lavandula dentata* (*fringed lavender*)	*Pseudomonas* sp., *Bacillus* sp.	IAA, HCN, siderophores, lipase, protease, cellulase, pectinase	plant growth promotion	[11]
30	*Lotus corniculatus* (*bird’s foot trefoil*)	*Serratia plymuthica*, *Pseudomonas mandelii*, *Tsukamurella pulmonis*	IAA, HCN, siderophores, cellulase	plant growth promotion	[119]
31	*Matricaria chamomilla* (*German chamomile*)	*Bacillus pumilus*	lipopeptides	antifungal	[99]
32	*Calendula officinalis*	*Bacillus mojavensis*, *Bacillus subtilis* subsp. *subtilis*, *Bacillus subtilis* subsp. *spizizenii*, *Bacillus endophyticus*, *Paenibacillus brasilensis*, *Paenibacillus polymyxa*, *Lysobacter enzymogenes*	-	antibacterial, antifungal, nematicidal	[108]
33	*Mentha longifolia* (wild mint)	*Bacillus* sp., *Bacillus aryabhattai*, *Bacillus pumilus*, *Bacillus megaterium*, *Bacillus toyonensis*	IAA, ACC deaminase, siderophores	plant growth promotion	[120]
34	*Mentha spicata* (spearmint)	*Bacillus anthracis*, *Bacillus toyonensis*	cellulase, xylanase, amylase, pectinase	enzymatical	[121]
35	*Polygonum cuspidatum*	*Bacillus safensis*	volatile metabolites (butanal, 3-methyl-, 2-heptanone, 6-methyl-5-methylene-, hydrogen azide, propene, 2-butene, 6-oxabicyclo3.1.0 hexane)	antifungal	[99]
36	*Oenothera biennis* (common evening primrose)	*Rhodococcus erythropolis*, *Rhizobium* sp.	IAA, HCN, cellulase	plant growth promotion	[119]
37	*Plantago lanceolata* (ribwort plantain)	*Pseudomonas brassicacearum*, *Bacillus methylotrophicus*	-	biocontrol, plant growth promotion	[107]
38	*Calendula officinalis*	*Bacillus halotolerans* Cal.l.30	surfactin, iturin, fengycin, bacillaene, bacillibactin, FAS-PKS, subtilosin A, bacilysin	antibacterial, antifungal, nematicidal	[122]
39	*Peperomia dindygulensis*	*Streptomyces* sp. YINM00001	cyclohexiamide, dinactin, warkmycin, anthramycin, alkylresorcinol, lanthipeptide, melanin, ectoine, geosmin	antibacterial, antifungal	[123]
40	*Origanum vulgare*	*Bacillus* sp.	paeninodin, terpene, paenilarvins	biocontrol	[124]
		*Paenibacillus* sp.	polymyxin, paenicidin A	antimicrobial	
41	*Mikania micrantha*	*Sphingomonas paucimobiliz*	Zeaxanthin	antioxidant	[105]
		*Micrococcus yunnanensis*	microansamycin, stenothricin	biocontrol	
42	*Mimosa pudica*	*Staphylococcus caprae*	Aureusimine, staphyloferrin A	Plant growth promotion	
		*Neobacillus drentensis*	Fengycin	biocontrol	
		*Priestia megaterium*	Surfactin, bacitracin, carotenoid	biocontrol	
43	*Millettia pachycarpa Benth*	*Paenibacillus peoriae* IBSD35	NRPSs, Fusaricidin synthetase, paenibacterin, gramicidin synthase	Biocontrol, antibiotic	[114]
44	*Panicum turgidum*	*Cellulosimicrobium* sp. JZ28	alkylresorcinol	Biocontrol	[125]
45	*Brassica napus* L.	*Pseudomonas fluorescens* BRZ63	alginate, LPS, siderophore, bacterioferrin, tryptophan, PQQ cofactor	biocontrol, enzymatic, plant growth promotion	[126]
46	*Suaeda fruticosa*, *Suaeda mollis*, *Mesembryanthmum nodiflorum*, *Arthrocnemum indicum*	*Bacillus albus* strains	petrobactin, bacitracin, thuricin	Biocontrol	[127]
47	*Bacopa monnieri*	*Bacillus* sp. LCF1	surfactin, iturin, fengycin, type I PKS	Biocontrol	[128]
48	*Arnica montana* L.	*Streptomyces* sp.	siderophore, ectoine, terpene, bacteriocin, butyrolactone, lantipeptide, melanin, NRPS, PKS, cyclohexiaminde	biocontrol, enzymatic, plant growth promotion	[128]
49	*Echinacea purpurea*	*Rheinheimera* sp. RS3	Resorcinol, lantipeptide, hserlactone, bacteriocin, NRPS	Antimycobacterial potential, biocontrol	

Note: LPS—lipopolysaccharide, NRPS—nonribosomal peptide synthetase, PKS—polyketide synthase.
